# Sinus arrest following right coronary artery stent implantation

**DOI:** 10.1186/1755-7682-5-11

**Published:** 2012-03-20

**Authors:** Peerawut Deeprasertkul, Ranjan K Thakur

**Affiliations:** 1 Division of Cardiology, Michigan State University, Lansing, MI, USA; 2 Clinical Medicine, Division of Cardiology, Michigan State University, Lansing, MI, USA; 3 Department of Cardiology, Sparrow Hospital, Michigan State University, 1215 E Michigan Ave, Lansing, MI 48912, USA

**Keywords:** Sinus, Arrest, RCA, Stent, Implantation

## Abstract

Sinus arrest rarely occurs during acute myocardial infarction involving the right coronary artery (RCA) and sinus node (SN) artery. We report a rare case of sinus arrest caused by SN artery occlusion following RCA stenting. A 56-year-old woman with a significant history of RCA stenosis with prior bare metal stenting, presented to the emergency department with anginal chest pain. Initial work up showed significant elevation of cardiac troponin T with T-wave inversion in the inferior leads on electrocardiogram (ECG). Coronary angiography revealed a 90% stenosis of midportion of the RCA, mild occlusion in the left anterior descending coronary and left circumflex coronary arteries. Stenting was performed on the RCA lesion. Immediately after undergoing those interventions, thrombosis developed and occluded SN artery. Electrocardiogram showed junctional escape rhythm without P waves at a heart rate of 30 beats per minute, suggesting sinus arrest. The clot in the SN artery was identified and thrombectomy was performed. Neither symptoms nor hypotension were identified during this arrhythmia. Six days later, normal sinus rhythm began to appear on EKG with improving heart rate, and patient still remained completely hemodynamically stable. Pre-discharge exercise stress test had shown 50% predicted heart rate without ST segment change. Sinus node dysfunction is commonly related to degenerative processes, and rarely caused by thrombosis in the SN artery. In our case, we emphasize the potential complication of sinus arrest after RCA stent implantation.

## Background

Sinus arrhythmias may occur during acute myocardial infarction involving the right coronary artery (RCA) [1,2]. They are usually transient sinus bradycardia and do not frequently cause hemodynamic instability [3]. We report a rare case of sinus arrest caused by sinus node (SN) artery occlusion following RCA stenting.

## Case presentation

**Figure 1:**
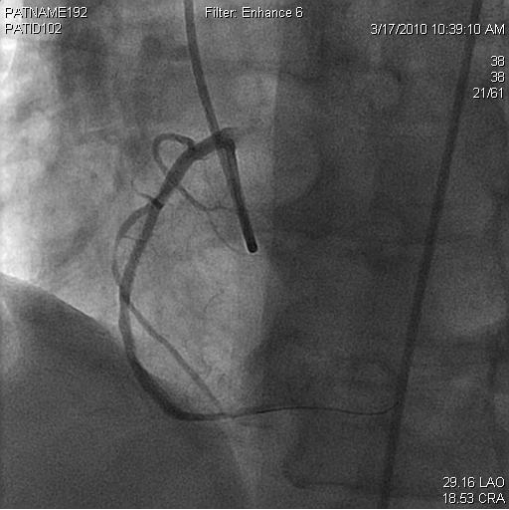
**The right coronary angiogram showing partial occlusion**.

**Figure 2:**
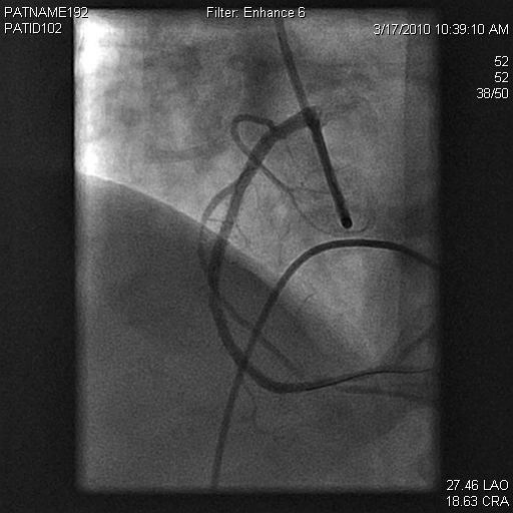
**The right coronary angiogram showing complete occlusion**.

**Figure 3:**
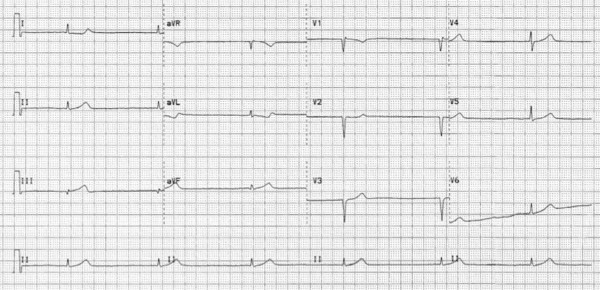
**Electrocardiogram showing sinus arrest with junctional escape**.

A 56-year-old woman with a significant history of RCA stenosis with prior bare metal stenting, hypertension, diabetes, dyslipidemia, smoking, and paroxysmal atrial fibrillation presented to the emergency department with anginal chest pain. Initial work up showed significant elevation of cardiac troponin T with T-wave inversion in the inferior leads. She subsequently underwent cardiac catheterization. Coronary angiography revealed a 90% stenosis of the midportion of RCA with patent stent at distal portion of RCA (Figure 1, 2) and only mild disease in left anterior descending coronary and left circumflex coronary arteries. An everolimus-eluting stent, 2.5 mm × 23 mm, was placed in the stenosis of midportion of RCA. Immediately after these interventions, thrombus developed and occluded SN artery. It originated from the proximal one third portion of RCA. Electrocardiographic monitoring showed junctional escape rhythm without P waves at a heart rate of 30 beats/min, suggesting sinus arrest (Figure 3). To prevent severe bradycardia and hypotension, transvenous temporary pacemaker was placed. The patient remained asymptomatic and without hypotension. The clot in the SN artery was identified and thrombectomy was performed. Within five minutes after intervention, Thrombolysis In Myocardial Infarction (TIMI) flow 3 was observed in the SN artery within 5 minutes and the transvenous temporary pacer was removed. Electrocardiogram revealed persistent junctional escape rhythm for 5 days after revascularization, with restoration of sinus rhythm on the sixth day. Heart rate showed a gradual improvement and the patient maintained a stable heart rate at 60 beats per minute. The patient remained hemodynamically stable without further need for temporary pacing. Pre-discharge exercise stress test had shown 50% predicted heart rate at 100 beats per minute, without ST segment change.

## Discussion

Sinus node dysfunction is usually related to degenerative processes, and is rarely caused by thrombosis in the SN artery [1]. Among sinus dysarrhythmia related to myocardial infarction, sinus arrest is rarely found in clinical practice and only a few cases have been reported. The ischemic insult can cause dysfunction of the sinus node [1]. However, the complete termination of sinus activity is rarely seen in the patient with acute coronary syndrome due to the sinus node's relative resistance to ischemic insult [1]. There are few case reports of sinus arrest following RCA intervention related to manipulation of RCA adjacent to SN artery [4,5]. Infrequently, patients required a permanent pacemaker [4]. In our case, developing thombus in SN artery occurred unexpectedly, despite the fact that the side branch of sinus node artery was quite far away from RCA stents. This could possibly be explained by thrombogenic mediators originating from the site of RCA clot, that were introduced in SN artery during intravascular catheter withdrawal. Coronary stent implantation can treat intravascular thrombus occlusion of major vessels; however, a side branch is often not protected [2]. In this case report, we emphasize the potential complication of sinus arrest after RCA stent implantation. This phenomenon should not be overlooked, and may need particular attention, even though stented RCA lesions are not in vicinity to SN artery.

Written informed consent was obtained from the patient for publication of this Case report and any accompanying images. A copy of the written consent is available for a review by Editor-in-Chief of this journal.

## Abbreviations

SN: Sinus Node; RCA: Right Coronary Artery.

## Competing interests

The authors declare that they have no competing interests.

## Authors' contributions

PD collected data, reviewed article, drafted the manuscript. RT edited and revised the manuscript. All authors read and approved the final manuscript.
